# Supplementary material for: Patterns of Intron Gain and Loss in Fungi
Source: PLoS Biol. 2004 Nov 30;2(12):e422. doi: 10.1371/journal.pbio.0020422 (PMC532390; doi:10.1371/journal.pbio.0020422)
Supplement: Table S1 — Also available at http://genes.mit.edu/NielsenEtAl/. (4.3 MB ZIP). [file pbio.0020422.st001.zip › NielsenEtAl/html/6.html]

AN3577.1.NCU02734.1.MG04805.1.FG00440.1


```
 CLUSTAL W (1.82) Multiple Sequence Alignments - Introns Inserted


Sequence 1: NCU02734.1	319 aa
Sequence 2: FG00440.1	320 aa
Sequence 3: AN3577.1	323 aa
Sequence 4: MG04805.1	327 aa
Alignment Length: 353 aa
Number Identitical Residues: 117 aa
Alignment Score (without introns) 6465


MG04805.1 	MAST--SLLRRALFY-~----------------D1NVTYDLEDSVSVASKDAALESLLRH
NCU02734.1	MAT--SSVLRRALLYV1PASSQKFMTKSLSLASD~NITYDLEDSVTPANKQTARTQLRDH
FG00440.1 	MAARSSSIIRRSLLYV1PGSSQKMLTKSLGLSSD~NVTYDLEDSVTPSLKDTARNQLREH
AN3577.1  	MAAR--NTLRRALLYI1PGSSQRFITKSRSLTAD~CVAYDLEDSVTPHKKAEARSLVRRA
          	**:   . :**:*:*  ..::.   :.: . ::*  ::*******:   *  *   :   

MG04805.1 	LKSLEGRPQFIREVAARVNPVQTAQGSRELTAL~AALPQVDAILLPKVDSSRDVLEAAQL
NCU02734.1	LSSLSDKPASIGELAVRINAVSTPYALDDLTTL~ASAPNLDAIVIPKVNS------ASDL
FG00440.1 	ISSLKARPSGISELAVRINAVPTPFALSDLTTL~APLRHVDAVVVPKVNS------AADL
AN3577.1  	LDEP--APPSIRERAVRINSVDSGLALADLTEV0LKSPNLSTIVIPKVNS------ASDL
          	:..    *  * * *.*:*.* :  .  :** :     ::.::::***:*      *::*

MG04805.1 	VDTADVERRDAFATLRQKQTGHGSDSNASGNYRRISLIALVESARAVEDLREICTALHGR
NCU02734.1	SFVTDVIR----HAAPQRHSSNP--TASP-----LKLLALIESARAVMDLRSICAASP--
FG00440.1 	TFVTDVLR----HVAPERHTAD---AGNP-----IKIIALIESARAVMDLSQICKASP--
AN3577.1  	TFVNDVIT----HTLAQQEAQGLPVTKSP-----ISLLALVESAKSLTNLTSITSATP--
          	  . **       .  ::.:   . :  .     :.::**:***::: :* .*  *    

MG04805.1 	KSDSGEPLAQLSGIVFGAEDFARDLSITRTPSLAEFQYARSKMVTAARAYDVPSIIDLVC
NCU02734.1	---------YLSGLIFAAEDFALDLSITRTPGLAEFLYARSAIVTAARAFDLPSSIDLVC
FG00440.1 	---------YLSGFIFAAEDFALDLSLTRTPSLTEFLYARSAIVTAARAAGLPSAIDLVC
AN3577.1  	---------LLQGLIFAAEDFALDLSLTRTPSLTEFLFARSAIATAARAANLPSTIDLVC
          	          *.*::*.***** ***:****.*:** :*** :.***** .:** *****

MG04805.1 	TAYKEPAR-------LEEECRNGRGMGFSGKQ~VIHPGQLETVNVLFGVSDAEAEWAVRI
NCU02734.1	TSYKGPEG----VKQLEEECQNGKEMGFNGKQ1CIHPSQVEVTQRMFAPAEAEVEWYVRI
FG00440.1 	TSYKGEQG----LKTLEEECAGGKSMGFNGKQ~CIHPSQVEVVQRMFAPDQREVEWAVRI
AN3577.1  	TTYKSNSGDGKPPAQLEEECRGGRGLGFNGKQ~CIHPSQVETVQRIYGPDEEEVNWAIRV
          	*:**    ....   ***** .*: :**.***  ***.*:*..: ::.  : *.:* :*:

MG04805.1 	VVANAKAEAQGRGAWAMDNQMIDAPVIGRAQGLVERARLCGIDVDALMEMWKDQQPE
NCU02734.1	LVADAQASAAGRGAWTLDGKMIDAPVVGKARAVVAKAAKCGMDIENIKAKWKDQKPE
FG00440.1 	SIGDEKASASGRGAWTLDGKMIDAPVVGKASAVIAKAKQCGIDVQSLRNTWKDQEPE
AN3577.1  	VVADEKAARQGRGAWTLDGKMIDVPVAEKAKAIVRKAEDCGVDVGALRAKWSGQEPE
          	 :.: :*   *****::*.:***.**  :* .:: :*  **:*:  :   *..*:**
```
